# Comparative Analysis of the *Brassica napus* Root and Leaf Transcript Profiling in Response to Drought Stress

**DOI:** 10.3390/ijms160818752

**Published:** 2015-08-11

**Authors:** Chunqing Liu, Xuekun Zhang, Ka Zhang, Hong An, Kaining Hu, Jing Wen, Jinxiong Shen, Chaozhi Ma, Bin Yi, Jinxing Tu, Tingdong Fu

**Affiliations:** 1 National Key Laboratory of Crop Genetic Improvement, National Center of Rapeseed Improvement in Wuhan, Huazhong Agricultural University, Wuhan 430070, China; E-Mails: liuchunqingaixin@126.com (C.L.); sjg301@163.com (K.Z.); anhong@webmail.hzau.edu.cn (H.A.); hukaining@gmail.com (K.H.); wenjing@mail.hzau.edu.cn (J.W.); jxshen@mail.hzau.edu.cn (J.S.); yuanbeauty@mail.hzau.edu.cn (C.M.); tujx@mail.hzau.edu.cn (J.T.); futing@mail.hzau.edu.cn (T.F.); 2 Key Laboratory of Oil Crop Biology and Genetic Breeding of the Ministry of Agriculture, Oil Crops Research Institute, Chinese Academy of Agriculture Sciences, Wuhan 430062, China; E-Mail: seedcq@263.net

**Keywords:** *Brassica napus*, drought stress, root, leaf, transcriptome, differentially expressed gene

## Abstract

Drought stress is one of the major abiotic factors affecting *Brassica napus* (*B. napus*) productivity. In order to identify genes of potential importance to drought stress and obtain a deeper understanding of the molecular mechanisms regarding the responses of *B. napus* to dehydration stress, we performed large-scale transcriptome sequencing of *B. napus* plants under dehydration stress using the Illumina sequencing technology. In this work, a relatively drought tolerant *B. napus* line, Q2, identified in our previous study, was used. Four cDNA libraries constructed from mRNAs of control and dehydration-treated root and leaf were sequenced by Illumina technology. A total of 6018 and 5377 differentially expressed genes (DEGs) were identified in root and leaf. In addition, 1745 genes exhibited a coordinated expression profile between the two tissues under drought stress, 1289 (approximately 74%) of which showed an inverse relationship, demonstrating different regulation patterns between the root and leaf. The gene ontology (GO) enrichment test indicated that up-regulated genes in root were mostly involved in “stimulus” “stress” biological process, and activated genes in leaf mainly functioned in “cell” “cell part” components. Furthermore, a comparative network related to plant hormone signal transduction and AREB/ABF, AP2/EREBP, NAC, WRKY and MYC/MYB transcription factors (TFs) provided a view of different stress tolerance mechanisms between root and leaf. Some of the DEGs identified may be candidates for future research aimed at detecting drought-responsive genes and will be useful for understanding the molecular mechanisms of drought tolerance in root and leaf of *B. napus*.

## 1. Introduction

Currently, 70% of total fresh water is used for agricultural consumption and a decrease of fresh water has caused serious environmental stress [1]. Rapeseed (*Brassica napus* L.) is one of the most important oil crops due to its edible oil and the meal that remains after oil extraction [2,3]. The growing threat of global warming and reduction in available fresh water have promoted the development of resistant rapeseed cultivars [4]. Plant root is an important tissue for the uptake of soil water and nutrients and for perceiving and transmitting soil water deficit signals to the shoot. Plant leaf plays critical roles in releasing water vapor through transpiration; especially in hot, dry, windy environments, water evaporates quickly. Under drought stress, to uptake enough water through the root and close stoma to avoid water loss through the leaf are critical to defend stress. When water uptake and water loss cannot keep balance by primary adaptive responses, different drought mechanisms through abscisic acid (ABA) and other signaling pathways, may be exploited to avoid and/or tolerate dehydration in root and leaf [5,6,7].

Various genes that function as stress sensors in signaling transduction pathways, which comprise a network of protein-protein reactions, transcription factors (TFs) and promoters, are activated in *Arabidopsis* and other plants [8,9]. Several pathways independently respond to environmental stresses through ABA dependent- and independent-manner, suggesting an extremely intricate gene regulatory network. The phytohormone ABA, which is triggered by stress, functions mainly in regulating plant water balance, stomatal closure, and coordinating the complex gene regulatory network enabling plants to cope with decreased water availability. Nearly 10% of the protein-coding genes in *Arabidopsis* were regulated by ABA [10]. The exogenous application of ABA also activates a number of genes that respond to dehydration stress [11]. Recently, a new model for ABA action, in which PYR/PYL/RCAR receptors function at the apex of a negative regulatory pathway to inhibit PP2C phosphatases and, in turn, directly increase SnRK2 kinases to trigger bZIP TFs and ABA-induced genes expression, has been proposed and validated [12,13,14,15]. This pathway is also critical for the direct control of guard cell physiology, which, in turn, regulates the stomatal response to stresses [16].

Most of the stress-inducible genes are dependent upon their interaction with TFs. As master regulators of gene transcription, TFs regulate activities of these factors, thereby employing or obstructing access of RNA polymerases to the DNA template [17]. In the *Arabidopsis thaliana* genome, approximately 1500 TFs are involved in stress responsive gene expression. Some of them are controlled by ABA but others are not [18]. ABA-dependent signaling systems regulons included: ABA-responsive element-binding proteins (ABRE), ABA binding factor (ABF), myeloblastosis related proteins (MYB), myelocytomatosis related proteins (MYC); While ABA-independent regulons are: APETALA2/ETHYLENE-RESPONSIVE ELEMENT BINDING FACTORS (AP2/EREBP), no apical meristem (NAM), *Arabidopsis* transcription activation factor (ATAF), and cup-shaped cotyledon (CUC) (NAC) and ZF-HD (zinc finger homeodomain) regulon [19]. In addition, several studies have identified the existence of function through both ABA-dependent and -independent pathways of stress response such as AP2/EREBP (ERF) family members [20].

As molecular responses, the interaction partners of the TFs determine the activation or repression of response pathways and are crucial to understand the regulatory networks that modulate plant defense responses, for example, AREB/ABF TFs and ABI5 can bind to ABRE, which is a major *cis*-acting element controlling ABA-responsive expression of the *Arabidopsis RD29B*, *COR78*, *LTI78*, and *RD20* gene; MYC and MYB TFs are shown to bind *cis*-elements MYCRS and MYBRS in the *RD22* promoter and co-operatively activate *RD22*; AP2/EREBP binds to DRE/CRT elements in the *RD29A* promoter; NAC TFs are identified as DNA-binding proteins interacting with *cis*-elements in *ERD1* promoter, analysis of which involved dehydration stress induction and in dark-induced senescence; many JA-inducible genes are target genes of *RD26*, which is a drought-inducible gene encoding a NAC TF [11]. WRKYGQK (WRKY) TFs have been shown to bind the TTGACC/T sequence (W-box) of the promoters of several important ABA-responsive or signaling genes, such as *ABI4*, *ABI5*, *ABF4*, *MYB2*, *DREB1A*, *DREB2A*, *RAB18*, *RD29A*, and *COR47* [21]. All these TFs function as transcriptional activators in the expression of stress-inducible genes [22].

Evaluations of the response of plant genotypes to drought stress have led to progress in the development of drought-resistant crops through both classical breeding efforts and modern genetic approaches [23,24,25,26]. Classical breeding approaches have revealed that stress-tolerance traits are mainly quantitative trait loci (QTLs), through which we have identified many stress-related genes, such as *Bo1*, a major boron tolerance QTL in bread (*Triticum aestivum*) [27], *Sub1*, a major submergence tolerance QTL in rice [28], *Vgt1*, a major flowering time QTL in maize [29] and the *Stg1*-*Stg4*, post-flowering drought-induced leaf senescence QTLs in Sorghum [30] and QTLs for water use traits in rapeseed [31,32]. However, the genetic selection of phenotypes response to drought stress is complex, ambiguous, and time-consuming; using classical breeding alone, even requires a broader interdisciplinary approach, involving an understanding of the factors. Recently, additional stress-inducible genes have been identified using transcriptomic analyses, such as microarray technology and ultrahigh-throughput RNA sequencing (RNA-seq). RNA-seq is a powerful technique that has been proven very useful for discovering many stress-inducible genes [33,34]. Recently, studies on the gene expression patterns of *Arabidopsis* [35,36], maize [37], wheat [38,39], barley [40,41], and rice [42,43] are common, and some have been performed on rapeseed stress [44], but the study on drought stress-responsive mechanism in *Brassica napus* (*B. napus*) has progressed slowly. In the present study, we performed large-scale transcriptome analysis of *B. napus* under air-dried dehydration stress using the Illumina sequencing technology for the purpose of obtaining a deeper understanding of the molecular mechanisms regarding *B. napus* responses to dehydration stress. Q2, a drought tolerant cultivar from Canada, was sampled at 0 and 24 h air dry treatment. We generated a total of more than 10 million reads from *B. napus* root and leaf, respectively. Then 6018 and 5377 genes were detected, differentially expressed in response to drought stress in root and leaf, respectively. The analyses showed that *B. napus* responded to dehydration mainly by down-regulating many biological processes, while those related to ABA signal transduction, phenylpropanoid biosynthesis, protection factors of macromolecules, and detoxification enzymes were highly activated. In addition, the two tissues of root and leaf showed distinctive differences in their gene expression profiles in response to drought stress. These sequencing datasets will serve as a valuable resource for candidate genes or markers that can be used to guide future efforts attempting to breed drought resistant *B. napus* cultivars.

## 2. Results and Discussion

### 2.1. Results

#### 2.1.1. Transcriptome Sequencing of Drought Stress

Q2 is characterized as drought tolerant *B. napus* pure line cultivar [45,46]. In this study, drought stress was imposed by initiating air dry down protocol starting 95 days after seeding (DAS) [47]. To obtain an overview of the transcriptome of *B. napus* root and leaf in response to drought stress, four cDNA libraries were constructed using the Illumina sequencing technology. These cDNA libraries were from root and leaf that had been exposed to air for 0 h (CK) and 24 h (DS). Compared to the well-watered control condition (Control Check) CK, the stress treatment showed until a loss of 75% of fresh weight (DS). A total of 11,957,048 CK root (CKR) raw reads, 12,476,345 drought stress root (DSR) raw reads, 12,338,528 CK leaf (CKL) raw reads and 12,054,260 drought stress leaf (DSL) raw reads were sequenced in libraries, respectively. After filtering out low-quality data (tags containing unknown nucleotide “N” or only adaptor tags) and single-copy tags, the final numbers of CKR (11,763,435), DSR (12,308,861), CKL (12,168,994) and DSL (11,905,805) clean tags were obtained. The results of the Illumina sequencing are shown as in Table 1.

**Table 1:** Illumina sequencing reads in CK root (CKR), drought stress root (DSR), CK leaf (CKL) and drought stress leaf (DSL) libraries.

Items	CKR	DSR	CKL	DSL
Total Raw Reads	11,957,048	12,476,345	12,338,528	12,054,260
Reads Containing N	22	29	28	22
Only Adaptors	115,945	81,690	84,941	68,814
Low Quality Reads	77,646	85,765	84,565	79,619
Clean Reads	11,763,435	12,308,861	12,168,994	11,905,805

CKR, root treated with water (air dry 0 h); DSR, root treated with drought stress (air dry 24 h); CKL, leaf treated with water (air dry 0 h); DSL, leaf treated with drought stress (air dry 24 h).

#### 2.1.2. Differential Gene Expression between the Root and Leaf Libraries

**Figure 1:**
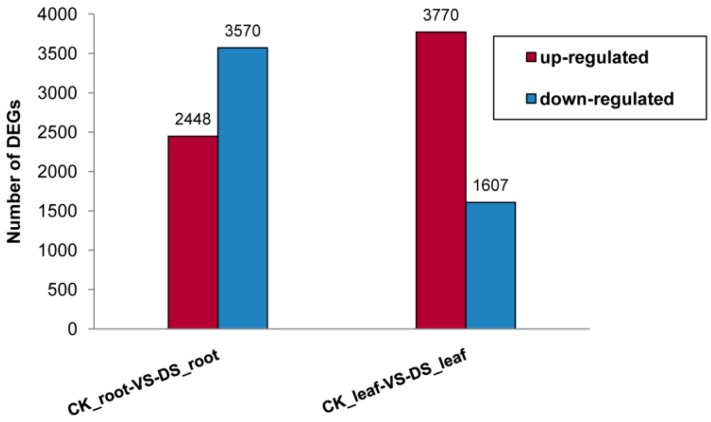
Changes in gene expression in Root and Leaf libraries. Numbers of up-regulated and down-regulated genes were summarized. CK_root-VS-DS_root refers to number of differentially expressed genes in root in response to drought stress; CK_leaf-VS-DS_leaf refers to number of differentially expressed genes in leaf in response to drought stress.

**Figure 2:**
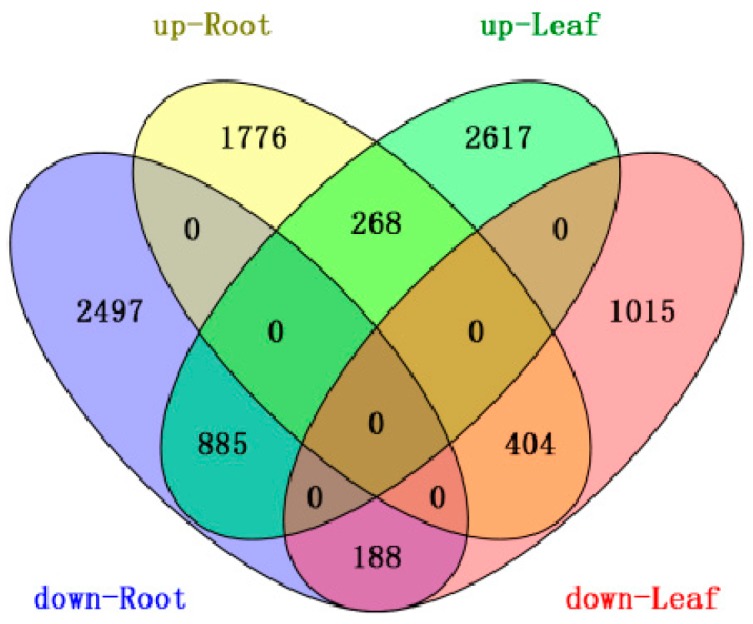
Overlap of differentially expressed genes in root and leaf in response to drought. Four-way Venn diagram showed the overlap of up- and down-regulated differentially expressed genes in root and leaf in response to drought stress. Regions corresponding to genes that are up-regulated commonly in both root and leaf are shaded in green. Regions corresponding to genes that are down-regulated commonly in both root and leaf are shaded in purple. Regions corresponding to genes that are up-regulated in leaf and down-regulated in root are shaded in blue. Regions corresponding to genes that are up-regulated in root and down-regulated in leaf are shaded in orange.

Based on “the significance of digital gene expression profiles” [48], a rigorous algorithm was developed to identify the differentially expressed genes (DEGs) in the two samples. The expression abundance of tag-mapped genes in the datasets was analyzed by counting the number of transcripts per million (TPM) clean tags. Through a comparison of our four Illumina libraries, a great number of differentially expressed transcripts were identified, and based on the above-described algorithm, 6018 DEGs of *B. rapa* (*Brassica rapa* L.) (Supplementary Data S1) were detected with significantly different expression levels in root, which included 2448 up-regulated and 3570 down-regulated genes. In addition, 5377 DEGs of *B. rapa* (Supplementary Data S2) were detected in leaf, which included 3770 up-regulated genes and 1607 down-regulated genes (Figure 1). This result showed the majority of the down-regulated genes were expressed in root, whereas leaf DEGs exhibited more up-regulated genes. In addition, we considered specific DEGs in root compared with leaf. A total of 3632 and 4273 unique DEGs were specifically expressed in root and leaf, respectively, and 1745 genes were identified in both root and leaf (Figure 2). Among the 1745 co-expressed genes, 268 and 188 genes were simultaneously up- and down-regulated in root and leaf; While 404 genes were specifically up-regulated in root and down-regulated in leaf, 885 genes were specifically down-regulated in root and up-regulated in leaf (Figure 2). Approximately 75% of co-expressed DEGs were conversely regulated between root and leaf samples, showing different expression patterns of DEGs in root and leaf existed in response to drought stress.

#### 2.1.3. Gene Ontology Function Analysis of DEGs

**Figure 3:**
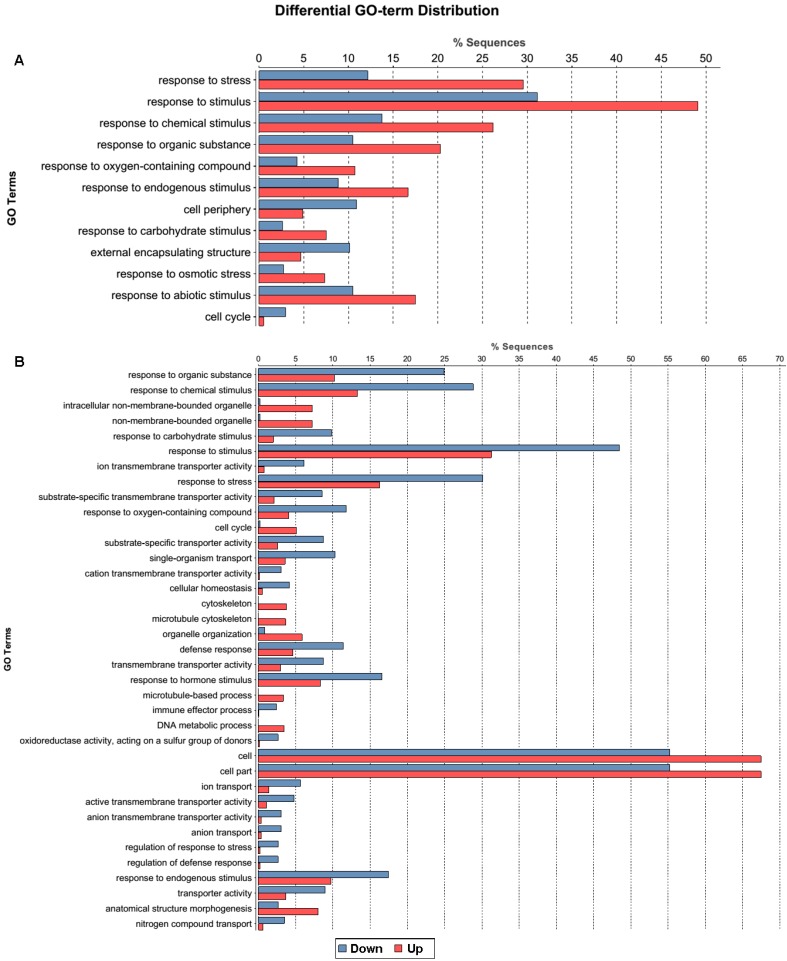
Statistical column diagram showing gene ontology (GO) annotation between up- and down-regulated differentially expressed genes (DEGs) in root (**A**) and leaf (**B**).

**Figure 4:**
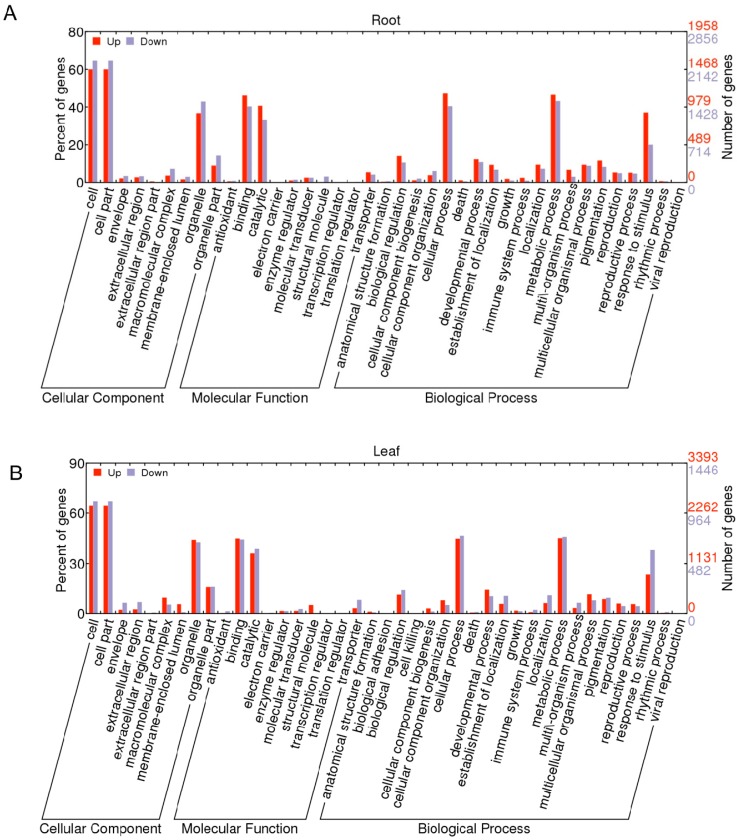
GO annotation of the differentially expressed genes in root (**A**) and leaf (**B**). The *x*-axis indicates the sub-categories, the left *y*-axis indicates the percentage of a sub-category of genes in that category, and the right *y*-axis indicates the number of unigenes in a sub-category.

Gene ontology (GO) is widely applied to understand the functional classification of differential gene expression data [49]. This analysis was performed to determine the major molecular functions, biological processes, and cellular components with which the DEGs were associated. A total of 6018 DEGs in root and 5377 DEGs in leaf of *B. rapa* were analyzed for GO category annotations using Blast2GO [50]. A GO term with *p* ≤ 0.001 and |log2| > 2 was defined as a significantly DEG-enriched GO term (Figure 3). Twelve GO terms were found as enriched biological processes based on the DEGs in root (Figure 3A): “response to stress”, “response to stimulus”, and “response to chemical stimulus”, which were the major GO terms. Simultaneously, more down-regulated genes were shown in “cell cycle”, “cell wall”, “external encapsulating structure” and “cell periphery” terms. However, 37 GO terms for the DEGs were found in leaf (Figure 3B): “cell”, “cell part”, and “organelle” were the major GO terms. Also comparatively higher contents of down-regulated genes were found in “response to stress” (63.0%), “response to chemical stimulus” (73.7%), and “response to stimulus” (52.7%) terms. Overall, most stress-related biological processes were up-regulated in root but down-regulated in leaf, and cellular components were mostly down-regulated in root but up-regulated in leaf. This GO terms enrichment analysis showed the critical biological processes in response to drought stress. We concluded from this result that *B. napus* seedlings mostly used the same terms and pathways, but different models of gene regulation were present in different tissue types in response to drought-stress conditions. In addition, GO (Gene ontology) classifications were also obtained to investigate the functions of the unigenes (Figure 4). A total of 6018 DEGs in root and 5377 DEGs in leaf were classified into 42 groups, and all could be categorized into three main classifications: “cellular component”, “molecular function”, and “biological process.” Among the biological process category, “cell” (approximately 59.3% in root and 60.8% in leaf) and “cell part” (approximately 59.3% in root and 60.8% in leaf) were the most dominant groups. GO enrichment analysis was also carried out in root (Supplementary Table S1) and leaf (Supplementary Table S2). Among the biological process category, response to stimulus process (about 44.6%) was the most dominant group in root, followed by response to stress (about 23.2%), biosynthetic process (about 23.2%), cellular biosynthetic process (about 22.6%), and response to chemical stimulus (about 22.1%). Regarding molecular functions, 12.3% of the unigenes were assigned to nucleic acid binding transcription factor activity, followed by phosphotransferase activity, alcohol group as acceptor (about 9.8%) and protein kinase activity (about 9.4%). In the cellular component category, intrinsic to membrane (12.8% for both) was the dominant group, followed by cell periphery (about 9.7%). In leaf, response to stimulus (about 44%) was also the most dominant group, followed by response to stress (about 23.6%), response to abiotic stimulus (about 16.2%), cellular component organization or biogenesis (about 13.7%) in the biological process category. Regarding molecular functions in leaf, 11.1% of the unigenes were assigned to nucleic acid binding transcription factor activity, followed by structural molecule activity (about 5.4%). In the cellular component category, membrane (about 36.3%) was the dominant group, followed by organelle part (about 24.3%), intracellular organelle part (about 20.8%).

#### 2.1.4. Pathway Analysis of the DEGs

**Figure 5:**
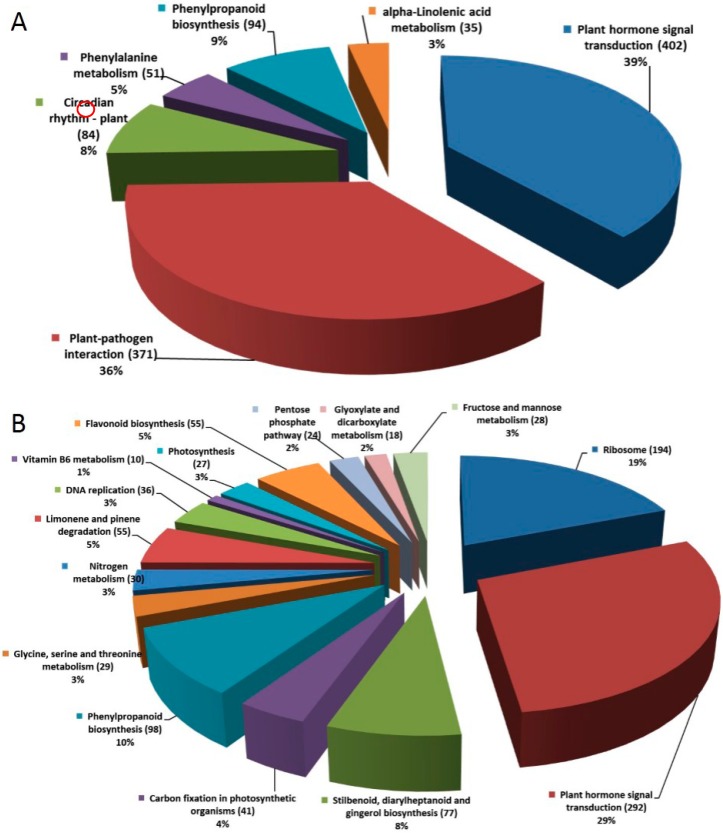
The summary enriched pathways of DEGs in root and leaf. (**A**) CK_root-VS-DS_root DEGs Pathway classification; (**B**) CK_leaf VS DS_leaf DEGs Pathway classification.

The DEGs were further analyzed according to their various biological functions. Pathway-based analysis was used to further understand the biological functions of the DEGs. The biological pathways of the DEGs were identified using KEGG (Kyoto Encyclopedia of Genes and Genomes) pathway analysis as well as the major public pathway-related database and included “metabolic”, “signal transduction”, “protein transport and degradation” together with “genetic information processing” [51]. The obtained pathways are listed in Supplementary Table S3. The significantly enriched pathways that the up-regulated and down-regulated genes were involved in, were identified using the same statistical algorithms as in the GO analysis (Figure 5). Among the significantly enriched pathways in root, “plant hormone signal transduction” (402 members for *B. rapa*) was the largest complex comprising several plant hormone patterns, including ABA, auxin (AUX), cytokinin, gibberellins (GA), ethylene (ET), brassinosteroid (BR), jasmonic acid (JA), and salicylic acid (SA). It followed by “plant-pathogen interaction” (371 members for *B. rapa*), “phenylpropanoid biosynthesis” (94 members for *B. rapa*), “Circadian rhythm-plant” (84 members for *B. rapa*) and so on, all of which had a *Q* value <0.01 (Figure 5A).

In the leaf libraries, the enriched pathways (*Q* value < 0.01) included “ribosome” (194 members for *B. rapa*), “plant hormone signal transduction” (292 members for *B. rapa*), “phenylpropanoid biosynthesis” (98 members for *B. rapa*) and so on (Figure 5B). This finding suggested the important drought tolerance pathways response to drought stress such as “plant hormone signal transduction” and “phenylpropanoid biosynthesis” which were consistent with previous results [52,53,54,55]. In addition, regulation of phenylpropanoid biosynthesis pathways is becoming increasingly associated with the MYB family of TFs, which control both biotic and abiotic stress responses [56].

#### 2.1.5. DEGs Encoding Transcription Factors

**Figure 6:**
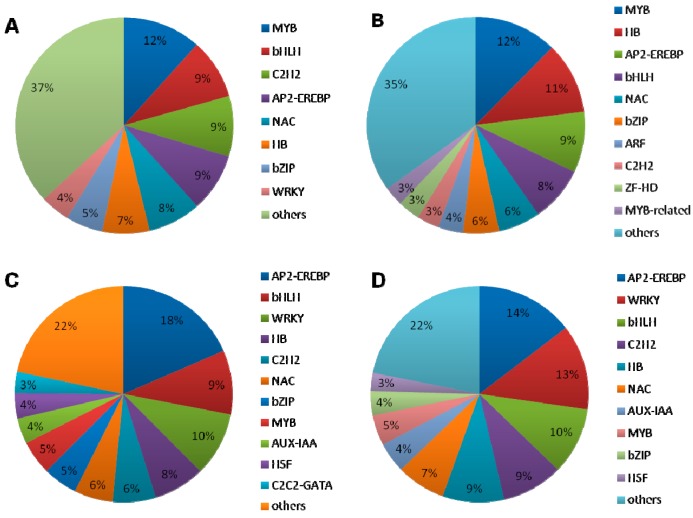
Distribution of transcription factor gene families in *B. napus*. (**A**) Distribution of transcription factors specially expressed in root; (**B**) Distribution of transcription factors specially expressed in leaf; (**C**) Distribution of transcription factors expressed both in root and leaf; (**D**) Distribution of transcription factors conversely expressed in root and leaf.

To assess the complex network of signaling pathways in drought stress, we further compared the expression profiles of the TFs in root and leaf. Genes (501 genes in root and 400 genes in leaf) encoding putative TFs that were responsive to drought stress in *B. napus* were identified based on a comparison with their expression of *Arabidopsis*, and 157 TFs were found to be co-expressed. In addition, 110 TFs (70% of the 157 co-expressed TFs) were conversely expressed in root and leaf. Of the root TFs, 344 TFs that were specifically expressed in root were divided into 16 groups based on a classification of their *Arabidopsis* homologs (Figure 6A). Also, five of the TF families which comprised 47% of these groups, including MYB (Bra027389, Bra039067, Bra020624, Bra018223), basic helix-loop-helix (bHLH) (Bra019773, Bra033690, Bra031721, Bra027501, Bra038792, Bra024115), C2H2 (Bra008445, Bra003582), AP2/EREBP (Bra037630, Bra006599, Bra009272, Bra026280), and NAC (Bra001596, Bra040152), played important roles in response to drought stress in root (Figure 6A). In addition, 243 TFs specifically expressed in leaf were also divided into 16 groups, in which four TF families, including MYB (Bra013000, Bra003443, Bra015911), HB (Bra024984, Bra028454), AP2/EREBP (Bra019777), and bHLH (Bra003073), accounting for 40% of the total TFs (Figure 6B). Four groups of TFs—AP2/EREBP (Bra024539, Bra010880, Bra022115, Bra024394), bHLH (Bra004489), WRKY (Bra008435, Bra035148, Bra003588, Bra005104), and HB (Bra027050, Bra007920)—were co-expressed in root and leaf (Figure 6C). Moreover, five groups—AP2/EREBP (Bra024539, Bra024394), WRKY (Bra008435, Bra035148, Bra003588, Bra005104), bHLH (Bra004489), C2H2 (Bra017432), and HB (Bra007920, Bra027050)—exhibited converse expressions between root and leaf (con-expression TFs) (Figure 6D). It has been demonstrated that MYB, NAC, C2H2, AP2/EREBP, bHLH, WRKY TFs participate in plant defense and play important roles in crosstalk among abiotic stress responses [19,57].

#### 2.1.6. Interaction between the Transcription Factors and Hormone-Related Genes

The interaction partners of these TFs activated as molecular responses are the key components of signal transduction pathways that take place during plant defense responses. These interactions determine the activation or repression of response pathways and are crucial in understanding the regulatory networks that modulate plant defense responses. The results showed that *ERD1* (Bra029193), *RAB18* (Bra031809, Bra037177), *RD29B* (Bra022585, Bra029121), and *RD20A* (Bra005501, Bra021847, Bra022936) were differentially expressed and had been proposed to function in the interaction with NAC and WRKY TFs and AREB/ABF, respectively [11]. In addition, MYC2 (Bra010178) was shown to be able to interact with all 12 of the JAZ proteins including JAZ1 (Bra025713, Bra016520, Bra031065), JAZ2 (Bra015880, Bra008172, Bra003778), JAZ3 (Bra022254, Bra021281), JAZ5 (Bra025977, Bra016604, Bra030986), JAZ6 (Bra016056, Bra008033), JAZ8 (Bra032362, Bra010794), JAZ9 (Bra016193, Bra007937, Bra003947), JAZ10 (Bra008846, Bra006190, Bra023399), JAZ12 (Bra002338, Bra020135), all of which were differentially expressed in our result and the mechanisms of interaction were similar to MYC4 (Bra013264) [19]. C2H2-type zinc-finger proteins ZAT10 (Bra032845, Bra010922) appeared to be partially dependent on ENA1/PMR2, a P-type ATPase required for Li^+^ and Na^+^ efflux in yeast [58].

Plants’ accumulations of phenylpropanoids and phenylpropanoid-based secondary products, like lignin, suberin, flavonoids, or condensed tannins, contribute substantially to all aspects of responses towards mechanical or environmental damage, such as drought, UV-light or wounding. In the present study, all encoding key enzyme transcripts in the phenylpropanoid biosynthetic pathway were found to be significantly up-regulated, including four *phenylalanine ammonia-lyase* (PAL) family genes: *PAL1* (Bra017210, Bra005221), *PAL2* (Bra039777, Bra006985, Bra003126), *PAL3* (Bra028793), *PAL4* (Bra029831); *cinnamate 4-hydroxylase* (C4H) (Bra022803, Bra022802, Bra021637, Bra021636, Bra018311); two isoforms of *4-coumarate: CoA ligase* (4CL): *4CL1* (Bra030429) and *4CL5* (Bra031263, Bra001819); *chalcone synthase* (CHS) (Bra036307), *dihydroflavonol reductase* (DFR) (Bra027457), and *flavonol synthase* (FLS) (Bra018076), suggesting these pathway genes might play important roles in *B. napus* response to dehydration. New information has been obtained on the interactions of TFs and *cis*-acting elements of several phenylpropanoid biosynthetic genes. R2R3-MYB and a bHLH factor, respectively, known to be one of the largest groups of TFs in our data, were together able to activate the CHS, FLS promoter; bZIP, and a R2R3-MYB factor work in conferring stress responsiveness [59,60]. The accumulation of these osmolytes could be critical in improving drought stress tolerance of *B. napus*.

Comprehensive molecular analyses have shown that the major and the best-known player, ABA, acting in concert with JA, ET, AUX, cytokinins, BR and SA, regulates the expression of many genes under osmotic stress conditions [12,16,61]. In the plant hormone signal transduction pathways, 402 DEGs in root and 292 DEGs in leaf were detected and most genes involved in ABA, JA, ET, BR and AUX. *PYR/PYL/RCAR* (Bra025048, Bra000938), *COI1* (Bra00500), *MPK6* (Bra004727), *BSK* (Bra020340) and *AUX1* (Bra031158, Bra000584) were differently expressed between the root and leaf. In addition, transcript levels of ion channels related genes Cl^−^, NO_3_^−^, malate^2−^ and K^+^ (Bra040606, Bra001068, Bra017567, Bra011367) were strongly induced by hormone signal. We hypothesized that elevated hormone levels caused the differently activated DEGs and then regulated downstream TFs, such as MYB, WRKY, bHLH, AP2/EREBP, C2H2 and so on, which in turn, regulated ion transports to stimulate stomatal closure in leaf and improve water uptake in root [17].

#### 2.1.7. Confirmation of Tag-Mapped Genes by qRT-PCR (Quantitative Real-Time PCR)

**Figure 7:**
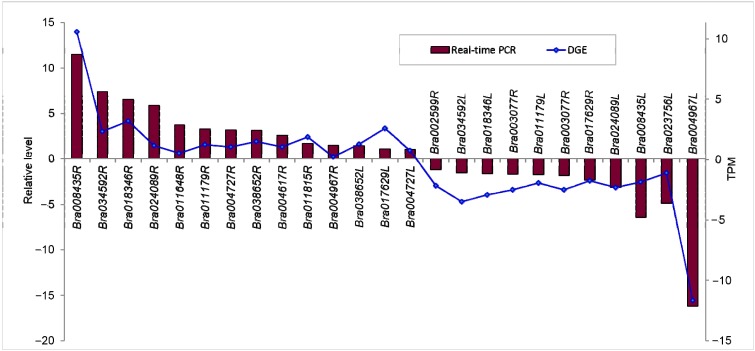
Real-time PCR validations of tag-mapped genes in root (R) and leaf (L). Relative level, (2^−Δ*C*t·Δ*C*t^); TPM, log2 Ratio (DS/CK).

To validate the Solexa/Illumina sequencing results, we performed quantitative RT-PCR on 25 selected DEGs after drought stress. Most of the real-time RT-PCR results were consistent with the DGE analyses (Figure 7), which demonstrated the reliability of the Illumina results. That DGE was more sensitive in the detection of low-abundant transcripts and small changes in gene expression than qPCR, might be the reason leading to a few inconsistent results between the transcriptome and qRT-PCR. Gene-specific primers of the selected genes were used for PCR analysis (Supplementary Table S4). In addition, five of the selected DEGs were also selected to perform quantitative RT-PCR in *B. napus* drought sensitive material 2021 root and leaf respectively [46] (Supplementary Figure S1). Some of the real-time RT-PCR results were consistent with the DGE analyses in drought tolerance material Q2, including *Bra011179R*, *Bra008435R*, *Bra008435L*, *Bra024089L*, *Bra002599R*. However, *Bra024089R*, *Bra011179L*, *Bra004727R*, *Bra002599L* were converse compared to the results in Q2. These data implied that most of the plants would defend the stress condition for survival in the same way, in which the differently expressed genes decided their different resistant cultivars.

### 2.2. Discussion

#### 2.2.1. Different Regulation Patterns between Root and Leaf Based on Transcriptome Profiles

The primary goal of this work was to identify a broad spectrum of drought-responsive genes in *B. napus* in order to estimate a range of physiological, metabolic, and cellular processes influenced by drought stress. This knowledge would allow characterization of the most tolerant pathways/genes associated with resistance to drought in *B. napus*. The tolerant inbred line Q2 had higher relative water content (RWC) and drought resistance index (DRI), lower relative electrical leakage [45]. Among various putative drought resistance mechanisms, the ability of plants to maintain relatively high tissue water potential and keep enzymes and ion homeostasis in the cells are critical relevant traits that influence yield under drought stress [62].

During deficits in the water supply, to improve root water uptake is one of the earliest responses to water deficit [63]. While most water loss occurs through the leaf via transpiration; the opening and closing of stomata are also closely related to drought stress [64]. It has been proposed that DEGs specific to the root are associated with osmotic stress, root hair, and relative root length, whereas DEGs specific to leaf are involved in stomata, water use efficiency (WUE) and leaf epicuticular waxes [65,66,67]. The central goal of this research was to identify a broad spectrum of drought-responsive genes and the expression patterns between root and leaf in *B. napus* to elucidate the different responsive mechanisms affected by drought stress.

For root and leaf analysis, a sequencing depth of 12.0 million tags was reached (Table 1), and more than 72% unique tags were matched with the genome and transcriptome of *B. rapa*, suggesting that the selected database was relatively complete. More than 70% of the genes had a match in the *B. rapa* database (Table 1), and the remaining 30% likely represented previously unidentified genes, partial and UTR sequences, non-coding RNA or sequences that were not conserved sufficiently to be identifiable across these species (*i.e.*, *B. napus versus B. rapa*) [68,69,70,71]. We analyzed the most differentially regulated tags, *i.e.*, those with a log 2 ratio >1 or <−1, a greater statistically significant value (*p* < 0.001) and false discovery rates (FDR < 0.001). Based on this rigorous algorithm, 6018 genes with significant differential expression levels were found for root (Supplementary Data S1) and 5377 genes were found for leaf (Supplementary Data S2). The number of DEGs in root was higher than that in leaf. Moreover, 3570 (59.3%) down-regulated DEGs were detected in root compared with 1607 (29.9%) down-regulated DEGs in leaf. In contrast to leaf, more down-regulated than up-regulated DEGs were shown in root suggesting the presence of different expression patterns in root and leaf.

A large gap in understanding still exists between the transcriptional control and the cellular execution during drought stress. These results may increase our understanding of the gene expression changes during drought stress. For example, through GO functional analysis, 92 categories were categorized using Blast2GO. DEGs involved in the responses to “chemical stimulus”, “osmotic stress”, “abiotic stimulus” and other stress stimuli were mostly up-regulated in root but down-regulated in leaf. Conversely, DEGs involved in “cell structure” and “organelle organization” were mostly down-regulated in root but up-regulated in leaf. Based on the DEGs among the transcriptome, more genes were down-regulated in the root, while more up-regulated genes were in the leaf during drought stress. It may be theorized that root played critical roles in directly tolerating drought stress and stimulus, such as the ability to undergo stress, and leaf played major roles in cell and structure-related effects, such as the ability to repair stress damages in order to sustain plant growth. This was consistent with a complementary division of labor by root and leaf in response to drought-stress conditions. Accordingly, drought stress signaling can be divided into three functional categories: ionic and osmotic stress signaling for the reestablishment of cellular homeostasis under stress conditions, detoxification signaling to control and repair stress damages, and signaling to coordinate cell division and expansion to levels suitable for the particular stress conditions [6]. Different mechanisms also achieved tolerance in two pairs of drought-tolerant and susceptible rice near-isogenic lines under drought stress through GO terms enrichment analysis and transcription factor gene families [5]. DEGs, transcription factor and ion distribution were also found to be different in the two Alfalfa lines corresponding to genotype-specific responses to salt [62].

The perception of drought stress activates the signal transduction cascades that interact with the baseline pathways that are transduced by phytohormones [72]. Previous results show a good agreement in the DEGs involved drought stress response and cross-talk in signal transduction pathways, such as ABA and GA [73], and sugar signaling (ABI4) [74]. Since ABA is the best-known stress hormone for triggering short-term responses, such as stomatal closure, and controlling longer-term growth responses, such as the maintenance of root growth, which stimulates water uptake [75]. ABA receptors, phosphatases type-2C (PP2Cs), and SNF1-related protein kinases2 are shown to control the ABA signaling pathway [12]. Primary phytohormone ABA receptor-PYR/PYL/RCAR (Bra025048, Bra000938) acts in concert with AUX-AUX1 (Bra031158, Bra000584), ET-MPK6 (Bra004727), BR-BSK (Bra020340) and JA-COI1 (Bra00500) to regulate stomatal closure through a complex network of signaling pathways [16,76,77,78]. The interaction between TFs and *cis*-elements plays important roles in crosstalk among abiotic stress responses [57].

#### 2.2.2. TFs Functional Protein-Protein Interactions in Hormone Transduction Pathway

A set of genes that are considered to be important regulators of drought-suffered plant, encoding components of signaling pathways or TFs, has been implicated in this process [6,11]. In the present study, MYB, bHLH, WRKY, AP2/EREBP, HB, and C2H2 were highly enriched during the drought-stress period, such as *MYB30* (Bra033067), *MYB60* (Bra018598), *MYB28* (Bra027389), *MYB108* (Bra040274, Bra001202), *bHLH92* (Bra033690, Bra027501), *ABA-INDUCIBLE BHLH-TYPE TRANSCRIPTION FACTOR* (*AIB*) (Bra039279, Bra004532), *ERF2* (Bra022115, Bra024954, Bra017495), *ERF4* (Bra027270, Bra021594, Bra001588), *WRKY40* (Bra035148, Bra008435, Bra003588), *WRKY18* (Bra011299, Bra023983, Bra010220), *WRKY33* (Bra005104, Bra017117, Bra000064), *WRKY57* (Bra038313), *HB40* (Bra017780), *HB7* (Bra039265), *C2H2-type zinc-finger proteins ZAT10* (Bra032845, Bra010922) and so on. All of them have been proposed to function in plant defense [79,80,81,82,83]. Among the DEGs for the highly expressed TFs, the MYB family contained the most abundant DEGs (12% in root and leaf), while AP2/EREBP was the highest co-expressed and con-expressed TFs, which were 18% and 14%, respectively. This finding suggested that different MYB family members were triggered for root and leaf in response to drought stress, whereas the same AP2/EREBP family members played crucial roles in regulating drought stress tolerance in both root and leaf. The MYB TFs are functionally diverse and present in all eukaryotes, in which R2R3-MYB class are plant-specific and involved in response to abiotic and biotic stress such as *AtMYB30* (Bra033067), *AtMYB60* (Bra018598) and *AtMYB96* [19,84,85,86]. *MYB2* (Bra004473) was up-regulated in root, interacting with MYBR *cis*-elements in the promoter of ABA-inducible gene *RD22* in *Arabidopsis* to control the ABA-inducible salt and dehydration responsive genes [87]. However, *RD22* was not detected in our data, we supposed the expression of *RD22* was not simultaneous and might be later than MYB and MYC in response to drought stress. TFs expressions are early and emergency responses of stress and the interaction downstream genes are late and adaptive response [88]. *MYB108* (Bra040274, Bra001202) were activated in the root, and they interacted with genes in jasmonate signaling pathway, mediated by reactive oxygen intermediates from both biotic and abiotic stress agents [89]. AP2/EREBP TFs, *ERF2* (Bra022115, Bra024954, Bra017495) functioned as activators and *ERF4* (Bra027270, Bra021594, Bra001588) acted as repressors that down-regulated basal transcription levels, which were shown to activate the defense pathway by interacting with the G-box in the promoter of *RD29A* and PR (pathogen-related) genes [90]. In addition, a high percentage of WRKY TFs were included in the con-expression TFs, suggesting that WRKY family members might also play an important role in the converse expression patterns. *WRKY* genes have been found to be up-regulated in response to pathogens, wounding, and senescence [79]. In our result, all of the *WRKY40* (Bra035148, Bra008435, Bra003588), *WRKY18* (Bra011299, Bra023983, Bra010220), *WRKY33* (Bra005104, Bra017117, Bra000064), *WRKY57* (Bra038313) were up-regulated in root but down-regulated in leaf, which were consistent with the result of the con-expression patterns between root and leaf (Figure 6D). Through the yeast one-hybrid assay, WRKY18, WRKY60, and WRKY40 were shown to interact with the W-box in the promoters of *ABI4* and *ABI5* genes and repressed *ABI4* and *ABI5* expression as negative ABA signaling regulators [91]. Activated expression of *WRKY57* conferred drought tolerance by elevation of ABA levels in *Arabidopsis*. ChIP assays demonstrated that WRKY57 could directly bind the W-box of *RD29A* and *NCED3* promoter sequences [92]. In the present study, the *NCED3* genes (Bra027336, Bra021558) were activated in both root and leaf and they were also the key genes in ABA biosynthesis [93]. NAC TFs are one of the largest plant-specific TFs and have been shown to function in relation to variety of abiotic and biotic stress responses [94]. Using the yeast one-hybrid system, three NAC members, ANAC019, ANAC055, and ANAC072 bound to the promoter of the *ERD1* and enhanced tolerance by inducing stress-related genes expression [95]. In our data, *ANAC019* (Bra018998), *ANAC055* (Bra001586), *ANAC072* (Bra026353, Bra019052), and *ERD1* (Bra029193) were up-regulated in root, consistent with a previous result. In addition, *NAC103* could induce reactive oxygen species (ROS) accumulation and cell death in *B. napus* [96] and overexpression of *BnNAC2* and *BnNAC5* genes in yeast remarkably enhanced the cells sensitive to high-salinity and osmotic stresses through inducing *COR15A* and *RD29A* expression [97]. The well-established phenylpropanoid biosynthetic pathway served as a useful model for studying metabolic regulation. The best characterized elements, G-box and H-box, were conserved and recognized by MYB, bHLH, or bZIP proteins. Cotransfection analysis demonstrated MYB12 strongly activated the promoters of *CHS*, *F3H*, *FLS*, and, to some lesser extent, the *CHI* promoter, the gene products of which were indispensable for the biosynthesis of phenylpropanoid [59]. MYB305 bound to the H-box in the promoter of *PAL2* and activated *PAL2* expression in tobacco protoplasts [60]. There was a hypothesis that the bZIP factors acted specifically in stimulus-dependent gene activation, while bHLH factors served to activate genes in a cell-identity dependent manner. Both factors would interact with MYB-like factors. Collectively, our data enables us to investigate the mechanism underlying gene expression in response to drought stress and the complex complementary division of labor by root and leaf in response to drought-stress conditions and strength, a hypothesis drought-related model previously proposed [89].

#### 2.2.3. Regulation Model for Root and Leaf under Drought Stress

**Figure 8:**
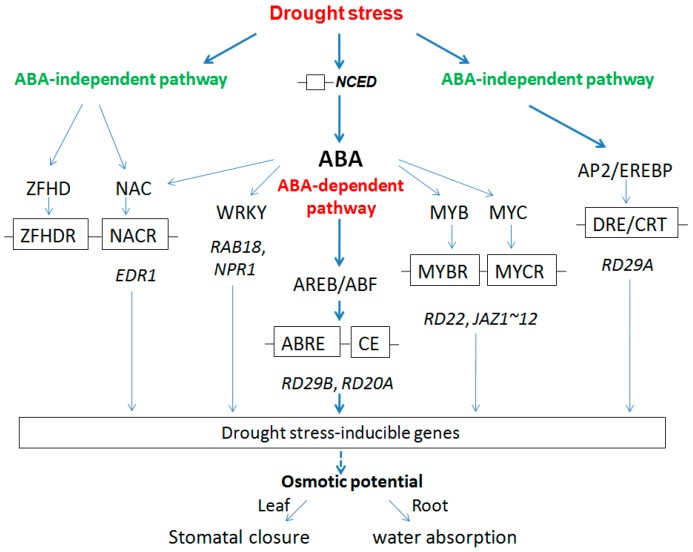
Proposed model for drought stress mechanism of root and leaf in *B. napus*.

The products of drought-induced genes can be classified into two groups. The first group includes proteins function to protect cells from stress through the production of important enzymes and metabolic proteins (functional proteins). The second group regulates signal transduction and gene expression in stress response (regulatory proteins) [98]. Functional proteins contained hydrophilic proteins including *dehydrins-RAB18* (Bra031809, Bra037177), *LTI29/ERD10* (Bra025819, Bra012230), *ERD14* (Bra008242, Bra003732); *osmotin-ATOSM34* (Bra033138), *proline synthesis enzyme-P5CS1* (Bra017051), *heat shock protein* (HSP)-*HSP70* (Bra038734, Bra001457), *HSP70T-2* (Bra005610), all of which were up-regulated except osmotin-*ATOSM34* (Bra033138), suggesting their functions in drought stress. Regulatory systems that are activated in response to drought stress have demonstrated the presence of both ABA-independent and ABA-dependent pathways. ABA-dependent signaling pathways that mediate stress adaptation are induced by at least two separate regulators and their interaction of *cis*-elements: AREB/ABF TFs and ABRE, MYB/MYC TFs and MYBR/MYCR, WRKY TFs and WRKYR (W-box). While ABA-independent pathways are: AP2/EREBP TFs and DRE/CRT, NAC/ZFHD TFs and NACR/ZFHDR. Subsequently, the down-stream drought-induced genes were activated, including *RD29B*, *RD20A*, *RD22*, JA-inducible genes (*JAZ1*-*12*), *RAB18*, *NPR1*, *RD29A*, *ERD1*. We hypothesized that these drought stress-inducible genes led to ion exchange, such as Cl^−^, NO_3_^−^, malate^2−^, and K^+^ in leaf and root that influenced the level of osmotic potential and then led to stomatal closure in leaf and improved water uptake in root. In addition, the elevation of the Ca^2+^ concentration as a result of the regulator of drought stress was another event that accompanied transcription factor which led to the changes of transports for ions such as K^+^, Cl^−^, and NO_3_^−^ and the subsequent change in turgor [16]. According to the above results, we now provide an overview about drought stress in a root and leaf regulated model in *B. napus* plants in Figure 8.

## 3. Experimental Section

### 3.1. Plant Materials and Stress Conditions

Seeds of *Brassica napus* Q2 were grown in the field in October 2010 for one month and then transferred into greenhouse conditions with a thermoperiod of 20 °C (day) and 18 °C (night) and a photoperiod of 16 h for two months . Prior to the treatment, roots were washed, carefully prevented from mechanical damage, and then placed in distilled water for 12–24 h. Both dehydration and control plants were placed under same growing conditions with 22 °C temperature, 65% relative humidity, and continuous light. For sampling of RNA-seq, six plants were exposed to air-drying on a filter paper for 24 h, and the control plants were kept further in distilled water. This treatment resulted in loss of 75% of fresh weight [46,47]. Leaves and roots samples were then collected from the control and dehydration-treated plants and immediately frozen in liquid nitrogen and stored at −80 °C until use.

### 3.2. RNA Isolation and Illumina Sequencing Results

Total RNA was isolated using the Total RNA Extraction kit (Bioteke, Bejing, China) according to the manufacturer’s instructions, and the RNA quantity and quality were monitored using RNA gel electrophoresis and a BioAnalyzer G2938A (Agilent Technologies, Waldbronn, Germany). A total of 10 μg RNA (≥400 ng/μL) was sent to Beijing Genomics Institute (Shenzhen, China) for Illumina sequencing, in which Illumina Gene Expression Sample Prep Kit, Illumina Sequencing Chip (flow cell) and the Illumina HiSeq™ 2000 System (Illumina, Inc., San Diego, CA, USA) were uses as the main reagents, supplies and instruments respectively. Libraries were prepared from a 17 bp (base pair) size-selected fraction following adapter ligation and agarose gel separation [99]. The original sequencing is deposited in SRA (Short Read Archive) with the accession number SRP045411.

### 3.3. Gene Expression Profiling RNA-Seq Analysis

After Illumina HiSeq™ 2000 sequencing, the raw reads were obtained and transformed into clean tags using certain data-processing steps. Then clean sequencing tags were aligned to the reference sequences, which covered all possible CATG+17-nt tag sequences of the genome and transcriptome of *B. rapa* using blastn, allowing only a 1-bp mismatch. Clean tags that mapped to reference sequences of multiple genes were filtered beside unambiguous clean tags. The number of unambiguous clean tags for each gene was calculated and then normalized to the TPM (number of transcripts per million clean tags) [99,100,101]. A rigorous algorithm was used to identify the differentially expressed genes (DEGs) between the four samples. The absolute values of |log 2 Ratio| ≥1 and FDR (False Discovery Rate) ≤0.001 were used as thresholds to judge the significance of differences in transcript abundance [48]. Then the DEGs were obtained and GO function and KEGG pathway analyses were carried out [51]. GO enrichment analysis provides all GO terms that significantly enriched in DEGs compared to the genome background, and filters the DEGs that correspond to biological functions. The calculating formula (1) is:

(1) $$P=1-\sum_{i=0}^{m-1}\frac{\left(\begin{array}{c} m\\ i \end{array}\right)\left(\begin{array}{c} N-M\\ n-i \end{array}\right)}{\left(\begin{array}{c} N\\ n \end{array}\right)}$$

where *N* is the number of all genes with GO annotation; *n* is the number of DEGs in *N*; *M* is the number of all genes that are annotated to the certain GO terms; *m* is the number of DEGs in *M*. The calculated *p*-value goes through Bonferroni Correction, taking corrected *p*-value ≤0.05 as a threshold. GO terms fulfilling this condition are defined as significantly enriched GO terms in DEGs. This analysis is able to recognize the main biological functions that DEGs exercise.

#### 3.4. qRT-PCR Analysis

To validate the results from the transcriptomic profile experiment, 25 selected DEGs from different functional categories were analyzed using qRT-PCR. Total RNA (160 ng) was reverse-transcribed using the RevertAid First Strand cDNA Synthesis Kit (Thermo, Hudson, NH, USA). The qRT-PCR reaction mixture was composed of the THUNDERBIRD SYBR qPCR Mix (TOYOBO, Osaka, Japan) and 2 μL of 50 × -diluted cDNA reaction mixture in a final volume of 20 μL with 200 nM of the gene-specific primers which were designed according to the reference unigene sequences using the Oligo 6 (Supplementary Table S4). The PCR reaction was performed using an iCycler iQ (BIO-RAD, Hercules, CA, USA) with the following cycle: denaturation at 95 °C for 1 min and annealing and polymerization at 60 °C for 20 s. Three biological repeats were performed, and actin was used as a reference gene.

## 4. Conclusions

The application of high-throughput sequencing technology has enabled us to detect DEGs in root and leaf under drought stress treatments at the rosette stage. In total, 6018 and 5377 DEGs were detected in root and leaf respectively. The number of down-regulated DEGs was higher in root than in leaf. The molecular functions of these genes were determined using GO, pathway-based and transcription factors and their interaction proteins analyses. These analyses showed that more stress-related biological process and relatively fewer cell component genes were triggered in root compared with leaf, indicating their complementary division of labor by root and leaf in response to drought stress conditions. The results provide a strong basis for future research on regulation patterns in root and leaf and their regulation mechanisms for drought stress, which could lead to improvements in drought-tolerant agronomic traits in *B. napus*. Future work should focus on characterizing these candidate target genes.

## Supplementary Materials

Supplementary materials can be found at http://www.mdpi.com/1422-0067/16/08/18752/s1.
