# Supplementary material for: Comparative Analysis of the Brassica napus Root and Leaf Transcript Profiling in Response to Drought Stress
Source: Int J Mol Sci. 2015 Aug 11;16(8):18752–77. doi: 10.3390/ijms160818752 (PMC4581270; doi:10.3390/ijms160818752)
Supplement: Supplementary file 1 [file ijms-16-18752-s001.zip › ijms-90233-Supplementary Information/ijms-90233-Supplementary Information.pdf]

Supplementary Information

Table S1. Significantly enriched gene ontology (GO) terms in differentially expressed genes (DEGs) for root.

**Significantly Enriched GO Terms for Root_Cellular Component**			
**Gene Ontology Term**	**Cluster Frequency**	**Genome Frequency of Use**	**Corrected *p*-Value**
intrinsic to membrane	489 out of 3832 genes, 12.8%	2498 out of 22,829 genes, 10.9%	0.01009
cell periphery	373 out of 3832 genes, 9.7%	1807 out of 22,829 genes, 7.9%	0.00083
external encapsulating structure	339 out of 3832 genes, 8.8%	1586 out of 22,829 genes, 6.9%	8.94×10^{-5}
cell wall	172 out of 3832 genes, 4.5%	697 out of 22,829 genes, 3.1%	8.22×10^{-6}
anchored to membrane	52 out of 3832 genes, 1.4%	188 out of 22,829 genes, 0.8%	0.02144

Table S2. Significantly enriched gene ontology (GO) terms in differentially expressed genes (DEGs) for leaf.

**Significantly Enriched GO Terms for Leaf_Cellular Component**			
**Gene Ontology Term**	**Cluster Frequency**	**Genome Frequency of Use**	**Corrected *p*-Value**
membrane	1289 out of 3554 genes, 36.3%	7696 out of 22,829 genes, 33.7%	0.04742
organelle part	862 out of 3554 genes, 24.3%	4933 out of 22,829 genes, 21.6%	0.00376
intracellular organelle part	738 out of 3554 genes, 20.8%	4058 out of 22,829 genes, 17.8%	6.22×10^{-5}
non-membrane-bounded organelle	397 out of 3554 genes, 11.2%	1336 out of 22,829 genes, 5.9%	2.73×10^{-39}
intracellular non-membrane-bounded organelle	397 out of 3554 genes, 11.2%	1336 out of 22,829 genes, 5.9%	2.73×10^{-39}
cell periphery	396 out of 3554 genes, 11.1%	1807 out of 22,829 genes, 7.9%	1.28×10^{-11}
external encapsulating structure	363 out of 3554 genes, 10.2%	1586 out of 22,829 genes, 6.9%	2.56×10^{-13}
ribonucleoprotein complex	263 out of 3554 genes, 7.4%	990 out of 22,829 genes, 4.3%	1.41×10^{-17}
nucleus	242 out of 3554 genes, 6.8%	1154 out of 22,829 genes, 5.1%	6.43×10^{-5}
nuclear part	238 out of 3554 genes, 6.7%	1119 out of 22,829 genes, 4.9%	2.50×10^{-5}
ribosome	237 out of 3554 genes, 6.7%	692 out of 22,829 genes, 3.0%	9.27×10^{-33}
nuclear lumen	224 out of 3554 genes, 6.3%	1026 out of 22,829 genes, 4.5%	6.89×10^{-6}
organelle lumen	224 out of 3554 genes, 6.3%	1026 out of 22,829 genes, 4.5%	6.89×10^{-6}
intracellular organelle lumen	224 out of 3554 genes, 6.3%	1026 out of 22,829 genes, 4.5%	6.89×10^{-6}
membrane-enclosed lumen	224 out of 3554 genes, 6.3%	1034 out of 22,829 genes, 4.5%	1.32×10^{-5}
extracellular region	197 out of 3554 genes, 5.5%	947 out of 22,829 genes, 4.1%	0.00137
ribosomal subunit	180 out of 3554 genes, 5.1%	467 out of 22,829 genes, 2.0%	7.28×10^{-32}
cell wall	163 out of 3554 genes, 4.6%	697 out of 22,829 genes, 3.1%	5.20×10^{-6}
large ribosomal subunit	97 out of 3554 genes, 2.7%	257 out of 22,829 genes, 1.1%	5.76×10^{-16}
cytoskeleton	86 out of 3554 genes, 2.4%	340 out of 22,829 genes, 1.5%	0.00038
small ribosomal subunit	83 out of 3554 genes, 2.3%	210 out of 22,829 genes, 0.9%	6.27×10^{-15}
microtubule cytoskeleton	74 out of 3554 genes, 2.1%	262 out of 22,829 genes, 1.1%	2.13×10^{-5}
anchored to membrane	66 out of 3554 genes, 1.9%	188 out of 22,829 genes, 0.8%	5.86×10^{-9}
chromosome	64 out of 3554 genes, 1.8%	219 out of 22,829 genes, 1.0%	3.93×10^{-5}
chromosomal part	58 out of 3554 genes, 1.6%	158 out of 22,829 genes, 0.7%	1.19×10^{-8}
chromatin	46 out of 3554 genes, 1.3%	87 out of 22,829 genes, 0.4%	1.43×10^{-13}

Table S2. *Cont.*

Significantly Enriched GO Terms for Leaf_Cellular Component			
**Gene Ontology Term**	**Cluster Frequency**	**Genome Frequency of Use**	**Corrected *p*-Value**
cytoskeletal part	24 out of 3554 genes, 0.7%	69 out of 22,829 genes, 0.3%	0.01246
microtubule	19 out of 3554 genes, 0.5%	51 out of 22,829 genes, 0.2%	0.02475
cytoplasmic microtubule	17 out of 3554 genes, 0.5%	45 out of 22,829 genes, 0.2%	0.04537

Table S3. KEGG (Kyoto Encyclopedia of Genes and Genomes) pathway results in root and leaf.

KEGG Pathway Results in Leaf						
**#**	**Pathway**	**DEGs with Pathway Annotation (3224)**	**All Genes with Pathway Annotation (22,126)**	***p*-Value**	***Q*-Value**	**Pathway ID**
1	Ribosome	194 (6.02%)	623 (2.82%)	1.14×10^{-26}	1.36×10^{-24}	ko03010
2	Plant hormone signal transduction	292 (9.06%)	1555 (7.03%)	1.37×10^{-6}	8.22×10^{-5}	ko04075
3	Stilbenoid, diarylheptanoid and gingerol biosynthesis	77 (2.39%)	316 (1.43%)	2.52×10^{-6}	9.29×10^{-5}	ko00945
4	Carbon fixation in photosynthetic organisms	41 (1.27%)	137 (0.62%)	3.10×10^{-6}	9.29×10^{-5}	ko00710
5	Phenylpropanoid biosynthesis	98 (3.04%)	456 (2.06%)	3.75×10^{-5}	9.00×10^{-4}	ko00940
6	Glycine, serine and threonine metabolism	29 (0.9%)	98 (0.44%)	0.000102	2.04×10^{-3}	ko00260
7	Nitrogen metabolism	30 (0.93%)	106 (0.48%)	0.00018923	3.24×10^{-3}	ko00910
8	Limonene and pinene degradation	55 (1.71%)	242 (1.09%)	0.00043356	6.50×10^{-3}	ko00903
9	DNA replication	36 (1.12%)	150 (0.68%)	0.00145221	1.94×10^{-2}	ko03030
10	Vitamin B6 metabolism	10 (0.31%)	26 (0.12%)	0.00240533	2.89×10^{-2}	ko00750
11	Photosynthesis	27 (0.84%)	111 (0.5%)	0.00438944	4.79×10^{-2}	ko00195
12	Flavonoid biosynthesis	55 (1.71%)	270 (1.22%)	0.00569307	5.69×10^{-2}	ko00941
13	Pentose phosphate pathway	24 (0.74%)	98 (0.44%)	0.00636964	5.88×10^{-2}	ko00030
14	Glyoxylate and dicarboxylate metabolism	18 (0.56%)	69 (0.31%)	0.00861442	6.99×10^{-2}	ko00630
15	Fructose and mannose metabolism	28 (0.87%)	122 (0.55%)	0.00873829	6.99×10^{-2}	ko00051
16	Phenylalanine metabolism	44 (1.36%)	219 (0.99%)	0.01555736	1.17×10^{-1}	ko00360
17	Base excision repair	22 (0.68%)	100 (0.45%)	0.02926962	2.07×10^{-1}	ko03410
18	Mismatch repair	23 (0.71%)	109 (0.49%)	0.04068947	2.50×10^{-1}	ko03430
19	Linoleic acid metabolism	10 (0.31%)	38 (0.17%)	0.04155532	2.50×10^{-1}	ko00591
20	Flavone and flavonol biosynthesis	18 (0.56%)	81 (0.37%)	0.04161865	2.50×10^{-1}	ko00944
21	Cyanoamino acid metabolism	32 (0.99%)	165 (0.75%)	0.05326877	2.91×10^{-1}	ko00460
22	Other glycan degradation	35 (1.09%)	183 (0.83%)	0.05342466	2.91×10^{-1}	ko00511

Table S3. *Cont.*

#	Pathway	DEGs with Pathway Annotation (3224)	All Genes with Pathway Annotation (22,126)	*p*-Value	*Q*-Value	Pathway ID
**KEGG Pathway Results in Leaf**						
23	Amino sugar and nucleotide sugar metabolism	48 (1.49%)	263 (1.19%)	0.05646386	2.95×10^{-1}	ko00520
24	Alanine, aspartate and glutamate metabolism	27 (0.84%)	137 (0.62%)	0.06033482	3.02×10^{-1}	ko00250
25	Biosynthesis of secondary metabolites	381 (11.82%)	2444 (11.05%)	0.07000647	3.24×10^{-1}	ko01110
26	Non-homologous end-joining	5 (0.16%)	16 (0.07%)	0.07127773	3.24×10^{-1}	ko03450
27	Steroid biosynthesis	25 (0.78%)	128 (0.58%)	0.07474566	3.24×10^{-1}	ko00100
28	Riboflavin metabolism	10 (0.31%)	42 (0.19%)	0.0756547	3.24×10^{-1}	ko00740
29	Lysine degradation	13 (0.4%)	59 (0.27%)	0.07980274	3.30×10^{-1}	ko00310
30	Glycosphingolipid biosynthesis—ganglio series	9 (0.28%)	38 (0.17%)	0.09165191	3.57×10^{-1}	ko00604
31	Glycolysis/Gluconeogenesis	41 (1.27%)	229 (1.03%)	0.09213469	3.57×10^{-1}	ko00010
32	Glycosaminoglycan degradation	10 (0.31%)	44 (0.2%)	0.09777733	3.67×10^{-1}	ko00531
33	Circadian rhythm—plant	47 (1.46%)	269 (1.22%)	0.1039593	3.78×10^{-1}	ko04712
34	Carotenoid biosynthesis	37 (1.15%)	209 (0.94%)	0.1183701	4.18×10^{-1}	ko00906
35	Synthesis and degradation of ketone bodies	3 (0.09%)	9 (0.04%)	0.1317673	4.52×10^{-1}	ko00072
36	Starch and sucrose metabolism	97 (3.01%)	601 (2.72%)	0.1478804	4.82×10^{-1}	ko00500
37	alpha-Linolenic acid metabolism	26 (0.81%)	145 (0.66%)	0.1510516	4.82×10^{-1}	ko00592
38	Plant-pathogen interaction	257 (7.97%)	1663 (7.52%)	0.1527424	4.82×10^{-1}	ko04626
39	One carbon pool by folate	8 (0.25%)	39 (0.18%)	0.1992956	6.13×10^{-1}	ko00670
40	Propanoate metabolism	14 (0.43%)	76 (0.34%)	0.2104047	6.25×10^{-1}	ko00640
41	Biotin metabolism	2 (0.06%)	6 (0.03%)	0.2134701	6.25×10^{-1}	ko00780
42	Fatty acid metabolism	13 (0.4%)	71 (0.32%)	0.22817	6.52×10^{-1}	ko00071
43	Nicotinate and nicotinamide metabolism	5 (0.16%)	24 (0.11%)	0.2657115	7.42×10^{-1}	ko00760
44	Anthocyanin biosynthesis	3 (0.09%)	13 (0.06%)	0.2917728	7.96×10^{-1}	ko00942
45	Peroxisome	27 (0.84%)	167 (0.75%)	0.309763	8.22×10^{-1}	ko04146
46	Glycerophospholipid metabolism	38 (1.18%)	240 (1.08%)	0.3149741	8.22×10^{-1}	ko00564
47	Phosphatidylinositol signaling system	28 (0.87%)	175 (0.79%)	0.3261654	8.33×10^{-1}	ko04070
48	Valine, leucine and isoleucine degradation	14 (0.43%)	85 (0.38%)	0.3538673	8.85×10^{-1}	ko00280
49	Phenylalanine, tyrosine and tryptophan biosynthesis	13 (0.4%)	79 (0.36%)	0.363401	8.90×10^{-1}	ko00400

Table S3. *Cont.*

**KEGG Pathway Results in Leaf**						
**#**	**Pathway**	**DEGs with Pathway Annotation (3224)**	**All Genes with Pathway Annotation (22,126)**	***p*-Value**	***Q*-Value**	**Pathway ID**
50	ABC transporters	39 (1.21%)	254 (1.15%)	0.3876352	9.23×10^{-1}	ko02010
51	Pentose and glucuronate interconversions	52 (1.61%)	342 (1.55%)	0.3920836	9.23×10^{-1}	ko00040
52	Homologous recombination	23 (0.71%)	149 (0.67%)	0.4168315	9.56×10^{-1}	ko03440
53	Ubiquinone and other terpenoid-quinone biosynthesis	15 (0.47%)	96 (0.43%)	0.427957	9.56×10^{-1}	ko00130
54	Selenocompound metabolism	6 (0.19%)	36 (0.16%)	0.4302276	9.56×10^{-1}	ko00450
55	Tyrosine metabolism	13 (0.4%)	84 (0.38%)	0.45346	9.89×10^{-1}	ko00350
56	Caffeine metabolism	1 (0.03%)	4 (0.02%)	0.4674023	1.00	ko00232
57	Nucleotide excision repair	27 (0.84%)	181 (0.82%)	0.4795134	1.00	ko03420
58	Indole alkaloid biosynthesis	8 (0.25%)	52 (0.24%)	0.4932763	1.00	ko00901
59	Sulfur metabolism	13 (0.4%)	87 (0.39%)	0.5070894	1.00	ko00920
60	Inositol phosphate metabolism	22 (0.68%)	149 (0.67%)	0.5087081	1.00	ko00562
61	Sphingolipid metabolism	11 (0.34%)	74 (0.33%)	0.5215932	1.00	ko00600
62	Pyruvate metabolism	23 (0.71%)	157 (0.71%)	0.5235224	1.00	ko00620
63	Benzoxazinoid biosynthesis	9 (0.28%)	62 (0.28%)	0.5598158	1.00	ko00402
64	Isoquinoline alkaloid biosynthesis	5 (0.16%)	34 (0.15%)	0.5649579	1.00	ko00950
65	Galactose metabolism	15 (0.47%)	105 (0.47%)	0.5754183	1.00	ko00052
66	Folate biosynthesis	4 (0.12%)	28 (0.13%)	0.5984961	1.00	ko00790
67	Ether lipid metabolism	9 (0.28%)	65 (0.29%)	0.6194095	1.00	ko00565
68	Glycerolipid metabolism	16 (0.5%)	115 (0.52%)	0.6197257	1.00	ko00561
69	Brassinosteroid biosynthesis	4 (0.12%)	34 (0.15%)	0.7505742	1.00	ko00905
70	Cysteine and methionine metabolism	24 (0.74%)	184 (0.83%)	0.7523371	1.00	ko00270
71	Arachidonic acid metabolism	4 (0.12%)	35 (0.16%)	0.7710338	1.00	ko00590
72	Pyrimidine metabolism	49 (1.52%)	371 (1.68%)	0.7937725	1.00	ko00240
73	Lysine biosynthesis	5 (0.16%)	45 (0.2%)	0.804655	1.00	ko00300
74	Valine, leucine and isoleucine biosynthesis	10 (0.31%)	85 (0.38%)	0.8110584	1.00	ko00290
75	Tropane, piperidine and pyridine alkaloid biosynthesis	7 (0.22%)	62 (0.28%)	0.8179297	1.00	ko00960
76	Regulation of autophagy	19 (0.59%)	156 (0.71%)	0.832077	1.00	ko04140
77	Terpenoid backbone biosynthesis	9 (0.28%)	80 (0.36%)	0.8423866	1.00	ko00900
78	Metabolic pathways	670 (20.78%)	4744 (21.44%)	0.8437551	1.00	ko01100
79	Fatty acid elongation	1 (0.03%)	12 (0.05%)	0.848979	1.00	ko00062
80	Thiamine metabolism	1 (0.03%)	12 (0.05%)	0.848979	1.00	ko00730
81	Zeatin biosynthesis	16 (0.5%)	136 (0.61%)	0.8547489	1.00	ko00908
82	beta-Alanine metabolism	8 (0.25%)	74 (0.33%)	0.8629269	1.00	ko00410

Table S3. *Cont.*

KEGG Pathway Results in Leaf						
**#**	**Pathway**	**DEGs with Pathway Annotation (3224)**	**All Genes with Pathway Annotation (22,126)**	***p*-Value**	***Q*-Value**	**Pathway ID**
83	Arginine and proline metabolism	18 (0.56%)	153 (0.69%)	0.866467	1.00	ko00330
84	Ascorbate and aldarate metabolism	17 (0.53%)	146 (0.66%)	0.8713508	1.00	ko00053
85	Pantothenate and CoA biosynthesis	6 (0.19%)	60 (0.27%)	0.8874153	1.00	ko00770
86	Glutathione metabolism	19 (0.59%)	166 (0.75%)	0.8986583	1.00	ko00480
87	Ubiquitin mediated proteolysis	54 (1.67%)	430 (1.94%)	0.898718	1.00	ko04120
88	Monoterpenoid biosynthesis	1 (0.03%)	16 (0.07%)	0.9195964	1.00	ko00902
89	Tryptophan metabolism	23 (0.71%)	205 (0.93%)	0.9328128	1.00	ko00380
90	RNA polymerase	16 (0.5%)	152 (0.69%)	0.9426849	1.00	ko03020
91	Biosynthesis of unsaturated fatty acids	8 (0.25%)	87 (0.39%)	0.9500522	1.00	ko01040
92	Histidine metabolism	3 (0.09%)	41 (0.19%)	0.9501572	1.00	ko00340
93	SNARE interactions in vesicular transport	12 (0.37%)	123 (0.56%)	0.956367	1.00	ko04130
94	Diterpenoid biosynthesis	9 (0.28%)	98 (0.44%)	0.9585866	1.00	ko00904
95	Glucosinolate biosynthesis	10 (0.31%)	107 (0.48%)	0.9596785	1.00	ko00966
96	RNA transport	71 (2.2%)	588 (2.66%)	0.9664407	1.00	ko03013
97	Fatty acid biosynthesis	6 (0.19%)	74 (0.33%)	0.9679602	1.00	ko00061
98	Other types of *O*-glycan biosynthesis	1 (0.03%)	22 (0.1%)	0.9687728	1.00	ko00514
99	Butanoate metabolism	5 (0.16%)	65 (0.29%)	0.9692734	1.00	ko00650
100	Photosynthesis—antenna proteins	2 (0.06%)	35 (0.16%)	0.9719417	1.00	ko00196
101	Porphyrin and chlorophyll metabolism	11 (0.34%)	121 (0.55%)	0.9735012	1.00	ko00860
102	Natural killer cell mediated cytotoxicity	10 (0.31%)	119 (0.54%)	0.984965	1.00	ko04650
103	*N*-Glycan biosynthesis	7 (0.22%)	93 (0.42%)	0.9872724	1.00	ko00510
104	Ribosome biogenesis in eukaryotes	30 (0.93%)	291 (1.32%)	0.9874966	1.00	ko03008
105	Phagosome	27 (0.84%)	271 (1.22%)	0.9905481	1.00	ko04145
106	Taurine and hypotaurine metabolism	1 (0.03%)	33 (0.15%)	0.9944895	1.00	ko00430
107	Purine metabolism	40 (1.24%)	388 (1.75%)	0.9949561	1.00	ko00230
108	Endocytosis	24 (0.74%)	264 (1.19%)	0.9972402	1.00	ko04144
109	Protein export	7 (0.22%)	113 (0.51%)	0.998366	1.00	ko03060
110	Citrate cycle (TCA cycle)	7 (0.22%)	115 (0.52%)	0.998683	1.00	ko00020
111	Circadian rhythm—mammal	1 (0.03%)	52 (0.24%)	0.9997252	1.00	ko04710
112	Basal transcription factors	4 (0.12%)	95 (0.43%)	0.9997404	1.00	ko03022

Table S3. *Cont.*

KEGG Pathway Results in Leaf						
**#**	**Pathway**	**DEGs with Pathway Annotation (3224)**	**All Genes with Pathway Annotation (22,126)**	***p*-Value**	***Q*-Value**	**Pathway ID**
113	Spliceosome	45 (1.4%)	486 (2.2%)	0.9998487	1.00	ko03040
114	RNA degradation	22 (0.68%)	291 (1.32%)	0.9999221	1.00	ko03018
115	Glycosylphosphatidylinositol (GPI)-anchor biosynthesis	15 (0.47%)	226 (1.02%)	0.999937	1.00	ko00563
116	Aminoacyl-tRNA biosynthesis	5 (0.16%)	129 (0.58%)	0.9999837	1.00	ko00970
117	Protein processing in endoplasmic reticulum	51 (1.58%)	603 (2.73%)	0.9999985	1.00	ko04141
118	Oxidative phosphorylation	14 (0.43%)	268 (1.21%)	0.9999997	1.00	ko00190
119	Proteasome	3 (0.09%)	134 (0.61%)	0.9999998	1.00	ko03050
120	mRNA surveillance pathway	13 (0.4%)	332 (1.5%)	1	1.00	ko03015

Table S4 Selected genes and corresponding primer sequences used for qRT-PCR (quantitative Real-Time PCR).

Gene Name	Primers (5'-3')	Used for
Bra008435R	CTCCCAAGAAACGCAA<br>GCAACTAACACGGACTGA	Real-time PCR
Bra034592R	TTTAGGAAGCAGAGCGAGCG<br>TTGAATGCTTTTTTGATAACGTCTG	Real-time PCR
Bra018346R	CTCCTGCGCCGTTAGTGCTA<br>GAAGATTGGTTAACGCGGAGAA	Real-time PCR
Bra024089R	TCTTCTTTGCGGCTCTTCTTG<br>TGTGACGGTTCCTGCTGAGTTA	Real-time PCR
Bra011648R	GTGCCGGCTTCGAGTTTCTT<br>CTCTGGAACGAGCTGCAGATTC	Real-time PCR
Bra011179R	ATTCCTCTTCTCAGCTCTCCTCG<br>CAGGTAGAAAGTTGTGACCGTGC	Real-time PCR
Bra004727R	CTGCTCAGCCATAGACACCCTTA<br>TCCATGAGCTCAAACACAACGTA	Real-time PCR
Bra038652R	GAGAGAGGTCGCAAGATTGATGA<br>ATCCACTCCACAAATCAGCTGC	Real-time PCR
Bra004617R	GTGGAGAAAGCCTCAATAGAAGATG<br>TGATCTTCGAGGTCTTGAGCAAC	Real-time PCR
Bra011815R	GCCGTTAAGTACATCGAAAGAGG<br>TAGTACCTTGCTTCGTCTTCGCT	Real-time PCR
Bra004967R	AGAGAGACTCGAGATCATGGACG<br>GCTGCAAGTCTCTTCCTTATCGT	Real-time PCR

Table S4. *Cont.*

Gene Name	Primers (5'-3')	Used for
Bra038652L	GAGAGAGGTCGCAAGATTGATGA<br>ATCCACTCCACAAATCAGCTGC	Real-time PCR
Bra017629L	GGGAAGACGAAGAAGAAACAGAA<br>AAGTAGAGAACCCATAACGAGCG	Real-time PCR
Bra004727L	CTGCTCAGCCATAGACACCCTTA<br>TCCATGAGCTCAAACACAACGTA	Real-time PCR
Bra002599R	GCGTCGTTTAGTGAGTTCAGCTT<br>AACCTCTCGCTCCTCAACTGC	Real-time PCR
Bra034592L	TTTAGGAAGCAGAGCGAGCG<br>TTGAATGCTTTTTTGATAACGTCTG	Real-time PCR
Bra018346L	CTCCTGCGCCGTTAGTGCTA<br>GAAGATTGGTTAACGCGGAGAA	Real-time PCR
Bra003077R	TCAACGCTCCTGTCCATATCG<br>TAGCTGGCAACCCTGATCTCA	Real-time PCR
Bra011179L	ATTCCTCTTCTCAGCTCTCCTCG<br>CAGGTAGAAAGTTGTGACCGTGC	Real-time PCR
Bra003077R	TCAACGCTCCTGTCCATATCG<br>TAGCTGGCAACCCTGATCTCA	Real-time PCR
Bra017629R	GGGAAGACGAAGAAGAAACAGAA<br>AAGTAGAGAACCCATAACGAGCG	Real-time PCR
Bra024089L	TCTTCTTTGCGGCTCTTCTTG<br>TGTGACGGTTCCTGCTGAGTTA	Real-time PCR
Bra008435L	CTCCCAAGAAACGCAA<br>GCAACTAACACGGACTGA	Real-time PCR
Bra023756L	ATACTGATCTGTGTTTCTCCGGC<br>AGAGGACAATGAAGGCGATGAACT	Real-time PCR
Bra004967L	AGAGAGACTCGAGATCATGGACG<br>GCTGCAAGTCTCTTCCTTATCGT	Real-time PCR

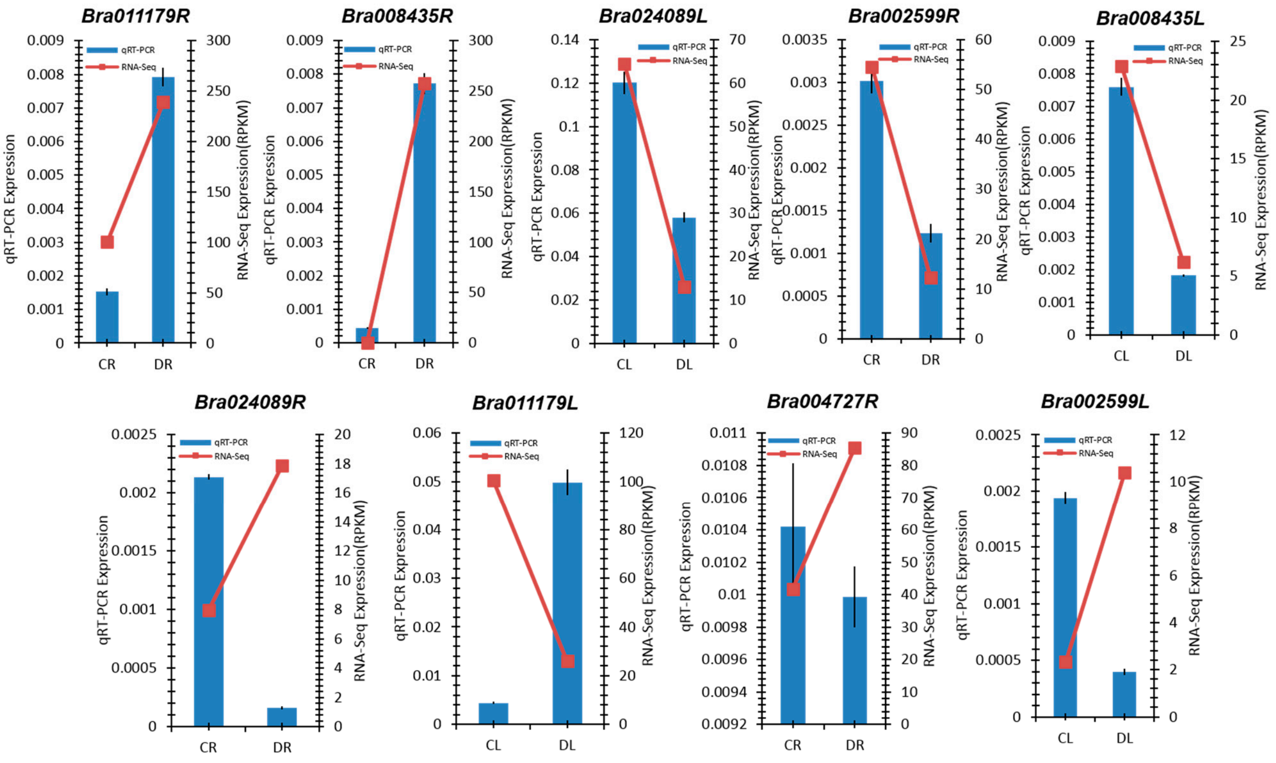

Figure S1. qRT-PCR (quantitative Real-Time PCR) verification of differentially expressed unigenes in drought sensitive material 2021. DR means drought stress in root; CR means control check in root; DL means drought stress in leaf; CL means control check in leaf. RNA-Seq expression (RPKM) is the Q2 RNA-Seq expression.
